# Genomes of *Helicobacter pylori *from native Peruvians suggest admixture of ancestral and modern lineages and reveal a western type *cag*-pathogenicity island

**DOI:** 10.1186/1471-2164-7-191

**Published:** 2006-07-27

**Authors:** S Manjulata Devi, Irshad Ahmed, Aleem A Khan, Syed Asad Rahman, Ayesha Alvi, Leonardo A Sechi, Niyaz Ahmed

**Affiliations:** 1 Pathogen Evolution Group, Centre for DNA Fingerprinting and Diagnostics, Hyderabad, India; 2 Centre for Liver Research and Diagnostics, Deccan College of Medical Sciences and allied hospitals, Hyderabad, India; 3 Department of Microbiology, Shri Shivaji College of Arts, Commerce and Science (SGB Amravati University), Akola, MS, India; 4 Cologne University Bioinformatics Centre, Cologne, Germany; 5 ISOGEM Collaborative Network on Genetics of Helicobacters, The International Society for Genomic and Evolutionary Microbiology, University of Sassari, Sassari, Italy

## Abstract

**Background:**

*Helicobacter pylori *is presumed to be co-evolved with its human host and is a highly diverse gastric pathogen at genetic levels. Ancient origins of *H. pylori *in the New World are still debatable. It is not clear how different waves of human migrations in South America contributed to the evolution of strain diversity of *H. pylori*. The objective of our 'phylogeographic' study was to gain fresh insights into these issues through mapping genetic origins of *H. pylori *of native Peruvians (of Amerindian ancestry) and their genomic comparison with isolates from Spain, and Japan.

**Results:**

For this purpose, we attempted to dissect genetic identity of strains by fluorescent amplified fragment length polymorphism (FAFLP) analysis, multilocus sequence typing (MLST) of the 7 housekeeping genes (*atp*A, *efp*, *ure*I, *ppa*, *mut*Y, *trp*C, *yph*C) and the sequence analyses of the *bab*B adhesin and *oip*A genes. The whole *cag *pathogenicity-island (*cag*PAI) from these strains was analyzed using PCR and the geographic type of *cag*A phosphorylation motif EPIYA was determined by gene sequencing. We observed that while European genotype (hp-Europe) predominates in native Peruvian strains, approximately 20% of these strains represent a sub-population with an Amerindian ancestry (*hsp-Amerind*). All of these strains however, irrespective of their ancestral affiliation harbored a complete, 'western' type *cag*PAI and the motifs surrounding it. This indicates a possible acquisition of *cag*PAI by the *hsp-Amerind *strains from the European strains, during decades of co-colonization.

**Conclusion:**

Our observations suggest presence of ancestral *H. pylori *(*hsp-Amerind*) in Peruvian Amerindians which possibly managed to survive and compete against the Spanish strains that arrived to the New World about 500 years ago. We suggest that this might have happened after native Peruvian *H. pylori *strains acquired *cag*PAI sequences, either by new acquisition in *cag*-negative strains or by recombination in *cag *positive Amerindian strains.

## Background

*Helicobacter pylori *is a Gram-negative bacterium that established itself in the human stomach possibly thousands of years ago [1]. This opportunistic pathogen infects over 50% of the worlds' population, causing no harm to most colonized people [2]. Only a small subset of infected people experience *H. pylori*-associated illnesses such as chronic gastritis, peptic ulcer disease, gastric carcinoma, and mucosa-associated lymphoid tissue (MALT) lymphoma. Associations of various clinical outcomes with disease-specific virulence factors remain dogmatic [3] years after the completion of genome sequences [4]. The debate has been further intensified as some studies have posed the possibility that *H. pylori *infection has some protective effects in esophageal diseases [3]. Also, possible symbiotic associations have been proposed based on the finding that *H. pylori *harbor protective, bacteriocin like effect and may therefore be beneficial to its host [5].

Subsequent to the decipherment of the potential of polymorphic DNA markers in reconstruction of human migration and phylogeography [6,7], pathogen genotypes were successfully used in tracking and analyzing patterns of human migrations [8-10] in different continents. Recently, sequence variation in *H. pylori *has provided a window into human population migration [11] and also revealed that impact of religions on stratification of human ethnic groups can be analyzed based on *H. pylori *haplotypes [12].

Ancient origins and dissemination of *H. pylori *are quite debatable in the context of the vast South American continent that has witnessed many different waves of population migration [13], especially in view of the fact that *H. pylori *has been present in this continent since pre-Columbian times [14]. However, evolution of virulence and fitness in such 'ancient' strains that arrived first in the Americas and then, possibly out-competed by the influx of 'modern' strains from Europe [14] remains largely unexplored.

A landmark study based on PCR based DNA motif analysis proposed that *H. pylori *jumped recently from animals to humans and, therefore, the acquisition of *H. pylori *by humans may be a recent phenomenon [15]. This study has been the basis for the idea of *'H. pylori *free New World' [15]. However, two independent studies based on large-scale analyses of candidate gene polymorphisms contrasted the idea of recent acquisition and suggest that *H. pylori *might have co-evolved with humans [11,16]. In view of these intriguing ideas on ancient origin of *H. pylori*, additional evidences based on strains from different geographical regions (especially those with a rich history of multiple waves of human migrations such as the South Americas) are clearly needed.

We attempted to dissect gene pool diversity of Amerindian isolates of *H. pylori *from Peru with an objective to explore ancient and modern features of the Peruvian *H. pylori *strains corresponding to different waves of human migration. We also looked if it is possible to link some of the native Peruvian strains to their ancestors in Asia.

## Results

### FAFLP based genotyping, candidate gene sequence analysis and multi-locus sequencing

**Figure 1:**
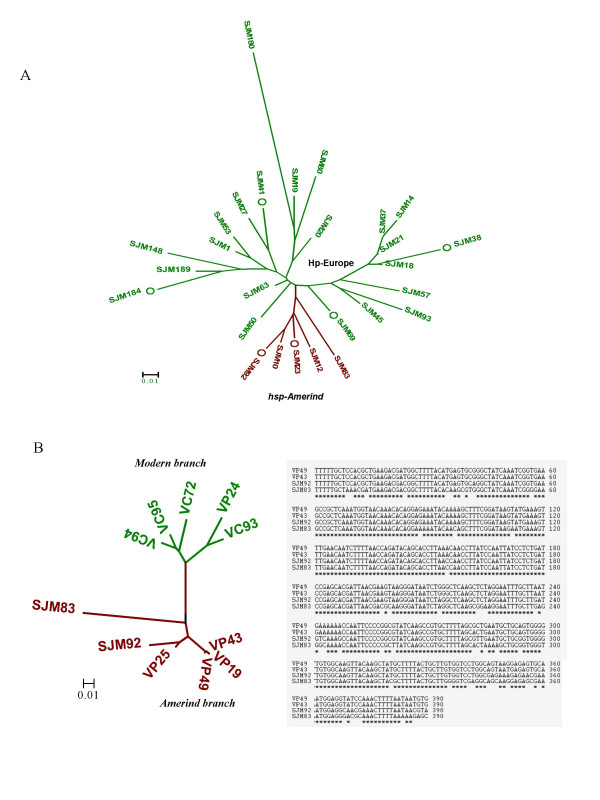
**A. **FAFLP analysis of *H. pylori *strains analyzed from native Peruvians (n = 27). The phylogenetic tree was developed based on various *amplitypes *generated for individual isolates after allele scoring and generation of similarity profiles in the form of binary tables. Genetic relationships in the form of a tree were deduced using MEGA 3.0 software using bootstrapping method at 10000 bootstrap trials. Two different lineages observed in the tree are colored as per the previous conventions [11, 20]. Six isolates from among those represented in the FAFLP tree (highlighted with circles) were also analyzed subsequently by MLST. **B. **Phylogenetic analysis of representative *hsp-Amerind *sequences from our isolates (SJM 83, 92) and those of other Amerind and non-Amerind (western) *H. pylori *sequences previously described by Ghose *et al*. from Caracas and Puerto Ayacucho in Venezuela [16]. SJM 83 and 92 formed a separate branch (maroon color) only with Amerind isolates (right). Both the Amerindian and western lineages (green color) are differentially colored as per previous conventions [11, 20]. Sequence alignment (left) of these Amerind sequences revealed significant conservation at the level of nucleotides.

Phylogenetic relationships assessed by FAFLP genotyping of Peruvian isolates (Figure 1A) revealed 2 different lineages corresponding to the hp-Europe and *hsp-Amerind*. Representative isolates from both the lineages obtained by FAFLP were subjected to reconfirmation by MLST analysis. We observed that all the isolates corresponding to the Amerind type FAFLP profile were found to be genetically closer to strains from Alaska and were therefore, accepted as genotype *hsp-Amerind*. All the other isolates from Peru clustered with those from Spain and therefore joined MLST genotype hp-Europe.

We looked at the geographic signatures of *oip*A gene sequences in Peruvian isolates and found that 7 of the 27 isolates including the 2 *hsp-Amerind *isolates had an Eastern type *oip*A (CT < 6) signature; 4 of these had a non functional gene (frame out) (Table 1). Nineteen of the Peruvian isolates had a western type *oip*A (CT ≥ 6), 15 of which had a functionally intact coding frame. It appears that *oip*A sequence conveys the European ancestry for a majority of these isolates, including some *hsp-Amerind*, however, *oip*A is a virulence linked gene and its resolution power for lineage identification is not as robust as that of the 7 housekeeping genes we used, or the *bab*B gene. It is likely that *oip*A might have been exchanged or recombined between different lineages (like most of the other virulence genes such as the *cag*PAI) to give hybrid genotypes.

The *bab*B gene sequences of our *hsp-Amerind *isolates were compared to the sequences previously reported for Amerindian isolates from Venezuela by Ghose *et al*. [16] by multiple sequence alignment and phylogenetic analysis (Figure 1B). These phylogenies, when studied in the light of MLST based inferences, revealed distinct affinities between Peruvian Amerindian strains and Venezuelan Amerind strains, pointing thus to their common Asian origins (Figure 1B).

In summary, our overall phylogenetic analyses, demonstrated that about 20% of the strains from native Peruvian patients that we examined share common genotypic patterns with Asian (Amerind) strains and therefore, Ancient Asian gene pool must have been the origin of the present day strains inhabiting the native Peruvians.

### Analysis of the cagPAI and the cagA gene in isolates from Spain, Peru and Japan

Overlapping primer amplification strategy to span entire *cag*PAI worked very well with our *hsp-Amerind *isolates (Figure 3A) where all the constituent genes of the PAI were successfully amplified. This indicates that the 5 Amerind strains we looked at represent a 'chimera genome' made up of an ancient like core genome component (MLST and *bab*B typing) and a modern type flexible genome component (*cag*PAI and its right junction typing). Tyrosine phosphorylation of the immunodominant *cag*A protein is known to occur at the EPIYA motifs at the C-terminus by the SRC family of kinases [17,18]. This EPIYA motif consists of 4 distinct EPIYA sites – EPIYA-A, -B, -C and -D, based on the amino acid sequence that neighbor it (Figure 3B). Based on the presence of these EPIYA motifs, *cag*A can be distinguished into the Western type (W) in case the -C site is present and the East Asian (EA) type in case where the -C site is replaced by the -D site. Our data for the type of EPIYA motifs present in Spanish (n = 6), Peruvian (n = 27), and Japanese (n = 16) strains revealed that the western type of *cag*A was predominant among all the Spanish and Peruvian strains. The Japanese isolates alone revealed East Asian type EPIYA D signatures (Figure 3B).

Results of the PCR of *cag*PAI from 26 Peruvian isolates studied in the present context have been depicted in Table 1. Briefly, the strains from Peru and Spain did not readily amplify regions of the *cag*PAI as did Japanese isolates, suggesting thereby a distinct allelic diversity at the primer binding sites in the western PAI of these isolates. This observation places native Peruvian strains closer to Spanish strains and therefore hints that both the Spanish and Peruvian strains harbor a similar type of *cag*PAI.

The right junction of the *cag*PAI also revealed similar acquaintances based on PCR based insertion deletion and substitution analysis of the region spanning *cag*A right junction to *glr*, for the 6 Peruvian isolates we analyzed. Such genotypes for all the isolates we used from Spain, Japan and Peru were determined earlier by Kersulyte *et al *[15]. Collectively, this study and the previous observations [15] demonstrated that the *cag *right junction motif types were shared by Spanish and Peruvian isolates and that Japanese isolates did not share genetic affinities with Peruvian strains. This observation again places native Peruvian strains closer to Spanish strains and therefore hints that both the Spanish and Peruvian strains share *cag*PAI insertion sites and the regions flanking them.

## Discussion

*H. pylori *is presumed to be an ancient colonizer of the human stomach which possibly co-evolved with its host. We report that at least some of the *H. pylori *strains found in Peru share considerable homology with strains found in Asia. This supports the hypothesis that *H. pylori *was associated with its host well before Asian people crossed the Bering strait (20,000 years BP) to colonize America. We also report that the *cag*PAI was acquired by native Peruvian strains probably from a European source. This might have occurred as a single import, most probably during the last 500 years of the spread of *H. pylori *in the South American continent. It is evident from the fact that the Amerindian Peruvian strains, though of an Asian descent, do not share characteristic features of Asian *cag*PAIs but show homology to the PAIs of European strains. Alternatively, the Amerindian strains might have gained the PAI through a series of recombination events over a period of time. But this hypothesis appears weak when we take into account the short time span of 500 years within which the European strains spread in the Americas.

We tried to potentate these ideas and to provide evidence in favor of the ancient origin of the pathogen and the possibility that some extraneous gene cassettes might have been acquired by otherwise symbiotic *H. pylori*, sometimes during its natural history. To support this proposition, our methodology was targeted with a two pronged approach to i) further substantiate ancient link of the pathogen and ii) to prove that the pathogenicity island was a 'recent' addition to the genome of *H. pylori*. Our analyses based on FAFLP and MLST linked about 20% of the Peruvian isolates to Amerindian genotype, and conveyed that *H. pylori *was most probably introduced to the New World by Asian people. We, therefore, disagree with the idea of an '*H. pylori *free New World' [15]. This disagreement of interpretation arose possibly because Kersulyte *et al*. [15] looked at only a few loci in the genome and stressed mainly on the motifs surrounding the *cag*PAI on its right junction.

**Figure 2:**
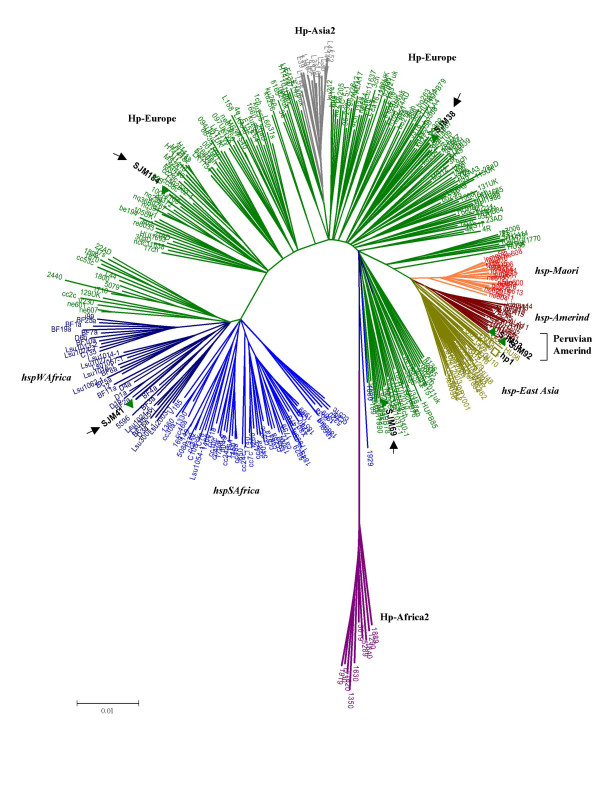
MLST analysis based on concatenated gene sequences of 7 housekeeping genes of *H. pylori *(Kimura-2 parameter). The phylogenetic tree was based on a total of 19 sequence records (concatenated) obtained under this study (SJM, HUPB, HU, CPY) while incorporating other ~400 sequence records from pubMLST database (pubmlst.org) which were specific to different genotypes in the world (Courtesy, Daniel Falush). Different genetic populations (Hp) and subpopulations (*hsp*) or genotypes are named and differentially colored after previous conventions [11, 20]. All SJM isolates, Amerindians (SJM23 and 92-highlighted) and non-Amerindians (arrowheads) analyzed by us from Peru are highlighted in bold face black fonts with green twigs indicating presence of a western type *cag*PAI.

**Figure 3:**
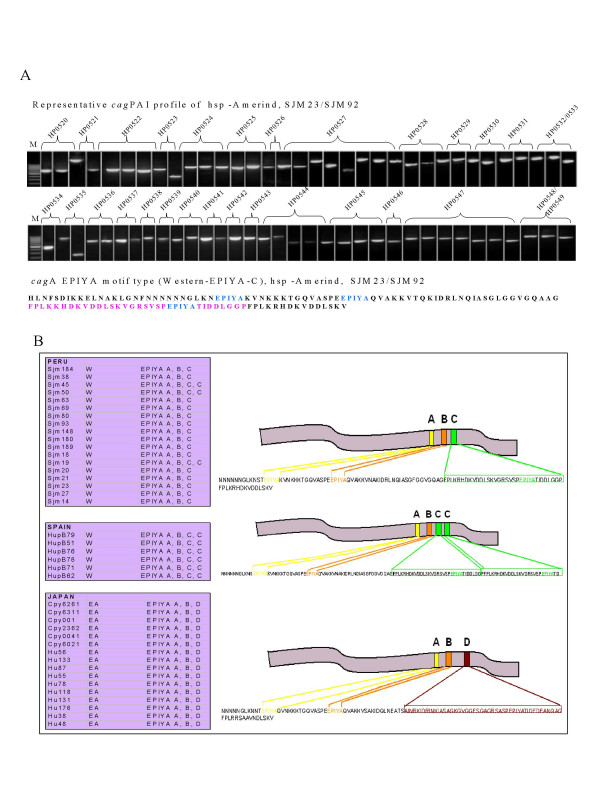
**A. **(Top) PCR based analysis of whole *cag*PAI of *hsp-Amerind *isolates from Peru. Overlapping primers spanning all of the constituent genes (see methods) amplified all the corresponding PCR products in the expected size range as described previously [30]. M indicates molecular weight marker (100 bp ladder). (Bottom) Amino acid signature of *cag*A (phosphorylation motifs – colored) – characteristic of modern *cag*A EPIYA (EPIYA C) as observed for all the SJM isolates from Peru. **B. **Pictorial depiction (right) of different types of *cag*A-EPIYA motif types prevalent in different *H. pylori *populations and their distribution in our isolates (boxes on the left).

We also looked at the *cag*PAI of such strains and found that the Peruvian isolates we tested carried western type (EPIYA C) *cag *islands (Figure 3B). We did not record any eastern type signature in the *cag*PAI (EPIYA D motif) in Peruvian isolates, given a distinct presence of ethnic Japanese in Peru. This inference also came from MLST data (Figure 2), where none of the Peruvian isolates clustered with Japanese genotype hp-EastAsia (Figure 2). Similarly, the absence of Asian/Amerindian type islands (or their remnants in native Peruvian isolates we analyzed, leads us to speculate that their ancestors in Asia were seemingly benign due to (natural?) absence of functional *cag *genes. This finding potentates the idea that *cag *genes in Peru mainly originated in Europe and therefore confirms the scenario proposed by Kersulyte *et al*. [15] as far as the *cag*PAI and its right junction is concerned.

Kauser *et al*., [19] from our group have previously described PCR analyses of the *cag*PAI for more than 300 strains from different parts of the world. Of these, the majority of strains from whom complete PAI was amplified were from Japan (57%) whereas only 18.6% strains from Peru and 13.3% strains from Spain could support amplification of an intact *cag*PAI, due mainly to allelic diversity present at the primer annealing sites. These observations were further endorsed by our present analysis that revealed that all the Peruvian isolates we analyzed carried only the European type PAI.

It has been recently reported that true Amerind strains either do not carry a *cag*PAI or carry only a vestigial, incomplete PAI [20]. It is already well known that *H. pylori *can import short stretches of DNA from strains from very different populations when they (presumably co-) infect individuals in the same location. However, it appears that the *cag*PAI and the region surrounding it had been exchanged by the Amerindian strains in Peru, where, the human population underwent major changes in recent history with the arrival of European conquerors and settlers. The long isolated Amerindian *H. pylori *strains thus came in contact with the European strains, which harbored a *cag*PAI. It is established that the *cag*PAI might give selective advantages during host colonization and therefore, "endogenous" Helicobacter, in Peru could be out-competed in a human community with newly arrived *cag*PAI positive strains introduced by the European conquerors. The "endogenous" Helicobacter strains could moreover acquire *cag*PAI during mixed infection with western *cag*PAI positive strains leading to the observed strains with Eastern-like core genome content and a western-type *cag*PAI. So this finding would really be interesting if it is clear that the entire *cag*-island had been exchanged. Then, there would be a very interesting question about the mechanism by which an entire island had been transferred; there is no doubt that such exchanges have happened at some point, but if they happened within the last 500 years, then, there would be much better chances of catching it in action. This is probably where the present data lead us to, and, we suggest that future efforts may be directed towards confirmation of this evolutionary mechanism.

Finally, it is possible that phylogenetic methods based on highly recombining gene loci [21-27] may not be fully perfect to predict genetic relationships in terms of inheritance from different ancestral populations, especially when we use tools such as Mega 3.0 [28] which do not support admixture analysis. Partly in view of this possibility and to ensure that our conclusions did not represent shortcomings of a single approach, we adopted an integrated genotyping strategy for the *cag*PAI [29,30] as well as the core genome through MLST of less recombining, neutral genes that encode cytoplasmic enzymes. Given the fact that these housekeeping genes are selectively neutral and uniform as compared to virulence associated loci such as the flagellins and *vac*A [31], recombinant and hybrid alleles that blur phylogeographic inferences, could be a rare occurrence rather than a rule. Nonetheless, it will be important to ascertain proportions of nearly pure and hybrid alleles among native Peruvian *H. pylori *through admixture analyses based on sophisticated population genetics tools [32] that reveal contemporary gene flow and proportion of different nucleotides inherited from ancestral populations on an evolutionary time-scale.

## Conclusion

In summary, our study based on profiling of several gene loci and neutral markers revealed certain distinctive genetic features of the *H. pylori *gene pool in Peru. Important among these features are a demonstrated genetic link with Asian (Amerindian) strains and the presence of an intact *cag*PAI with western type of gene motifs that point to recent acquisition of this important pathogenicity island. This indicates possible lateral gene transfers during colonization of Peruvian Amerindians with both 'ancient' and 'modern' strains for several generations. Such an admixed gene pool could be an important source of genetic information on pathogen evolution in real time to possibly understand how gene acquisition and loss on a population wide scale shape virulence and fitness in different pathogens.

This could also possibly provide for a reasonable model of geographic evolution to understand acquisition of virulence in pathogenic bacteria over a period of time.

## Methods

### Bacterial strains, genomic DNA and diagnostic PCR

Genomic DNA preparations for strains originating from Peru, Spain and Japan were provided by D. E. Berg and Asish Mukhopadhyay (Washington University, St. Louis, Mo.). These DNA were isolated from patients diagnosed with gastric ulcers from Spain (HupB); gastric cancer and DU cases from Japan (CPY, Hu); and from gastritis cases alone from Peru (SJM). However, in the current study, the clinical background of the individual isolates was not taken into account. The Peruvian isolates we looked at (n = 27) were originally from Native Peruvian people mainly of Amerindian ancestry from Lima [15]. PCR based analysis of genes namely *glm*M, *bab*B [16] and *oip*A was carried out to ascertain the quality of DNA samples we used. Also these PCR assays served as amplification level controls for the analysis of insertion, deletion and substitution in the *cag*PAI.

### Integrated genotyping of H. pylori based on chromosomal DNA signatures

Whole genome fingerprinting based on FAFLP genotyping was performed as described previously [21-23]. Briefly, the profiling of whole genome micro-restriction fingerprints with *Eco*RI/*Mse*I enzymes using fluorescence tagged primer pairs *Eco*RI+A/*Mse*I+0 and *Eco*RI+G or A/*Mse*I+0 was performed for all the strains. The PCR amplified fragments for each of the strains were then subjected to electrophoretic separation on a 5% acrylamide gel and scoring of the fluorescent markers was done using an automated DNA analysis workstation (ABI Prism 3100 DNA sequencer). Cluster analysis of DNA profiles was conducted on the basis of fingerprint characteristics. All the data obtained through molecular genotyping and DNA profiling were deposited in the genoBASE *pylori *database [33]. The genoBASE *pylori *server was queried for comparative analyses.

Genotyping based on candidate genes *oip*A and *bab*B was carried out as described [24-26]. Short stretches of *oip*A gene were analyzed to determine the 'geographic signature' based on CT repeat [27]. In addition, 600 bp region each from the 7 housekeeping genes spread throughout the genome [*atp*A, *efp*, *ure*I, *ppa *and *mut*Y, *trp*C, *yph*C] was amplified and sequenced for all the isolates exactly as described previously [11]. Sequencing was performed with both forward and reverse primers, using an ABI Prism 3100 DNA sequencer (Applied Biosystems, USA). PCR and direct sequencing were performed at least twice to determine and confirm the DNA sequences for each isolate. Consensus sequence for each of the samples was generated using Genedoc (version 2.6.002). Multiple alignments of sequenced nucleotides were carried out using Clustal X (version 1.81). Neighbor joining trees were constructed in Mega 3.0 [28], using bootstrapping at 10000 bootstrap trials (FAFLP and *bab*B) and through Kimura-2 parameters (for MLST). For construction of phylogenetic trees based on MLST genotyping procedures, sequences of 7 housekeeping genes of strains belonging to different established genotypes were obtained from the pubMLST database [34] (courtesy, Daniel Falush).

The nucleotide sequences of the 7 housekeeping genes for the 6 *hsp-Amerind *isolates we analyzed have been deposited in the GenBank [Accession numbers, GenBank:DQ462362–462367 (*atp*A), GenBank:DQ462368–462373 (*efp*), GenBank:DQ462374–462379 (*mut*Y), GenBank:DQ462380–462385 (*ppa*), GenBank:DQ462386–462391 (*trp*C), GenBank:DQ462392–462397 (*ure*I), GenBank:DQ462398–462403 (*yph*C)]. These and other sequences can also be requested from the authors.

### Profiling of the cagPAI

PCR analyses were carried out to find the status of the *cag*PAI using 8 sets of primers that amplified the *cag*A gene, its promoter region, the *cag*E and *cag*T genes and the left end of the *cag*PAI [25,26,29]. We also analyzed whole *cag*-PAI of the representative isolates (SJM92 and SJM23) from *hspAmerind *by PCR using overlapping primers as described by Blomstergren and colleagues [30]. The 3' end of the *cag*A gene was amplified using primers mentioned elsewhere [17] and the amplified products for strains from Spain, Peru, and Japan were sequenced with forward and reverse primers. The consensus sequences were then translated into amino acid sequences using GeneDoc software (version 2.6.002) and were then assigned to the Western or the East Asian group based on the C or D repeat present respectively in the EPIYA motif [18]. Chromosomal rearrangements are known to give rise to 5 types of insertion-deletion and substitution motifs in the region between the 3' end of *cag*A gene and the 3' end of the glutamate racemase (*glr*) gene. Although the statuses of these motifs for the Peruvian strains we analyzed were described previously by Kersulyte and colleagues [15], we re-assessed 6 of them by PCR exactly as described earlier [15].

## Authors' contributions

SMD and IA performed MLST typing and phylogenetic analysis. SMD also helped in analysis of *bab*B and *oip*A genotyping. AAK and AA performed PCR based analysis of the *cag*PAI genes. SAR extended bioinformatics  support. LAS provided expert clinical consultation and contributed to manuscript writing. NA performed FAFLP analysis, planned and supervised the study, wrote the manuscript and provided overall leadership. All the authors read and approved the final manuscript.

## Figures and Tables

**Table 1:** Characteristics of 26 Peruvian *H. pylori *isolates obtained from Amerindians and distribution, presence/absence and or rearrangement of various gene loci in their genomes; 0 – region not amplified, 1 – region present.

**Strains**	**Diagnostic PCR**		**Phylogenetic placement**			***oip*A *status***		***cagA genotype***		***cag-PAI spanning PCR***							
	
	*cagA*	*GlmM*	*FAFLP/MLST*	*babB*	*oipA*	No. of CT	Frame	*gene*	*EPIYA motif*	cagAP1	cagAP2	cagA1	cagA2	cagE	cagT	LEC1	LEC2
***A) Native Peruvian Strains***																	

SJM184	1	1	hp Europe	Modern	Eastern	5	Out	1	EPIYA A, B, C	1	1	1	1	1	1	1	1
SJM80	1	1	hp Europe	Modern	Western	6	Out	1	EPIYA A, B, C	1	1	0	1	1	1	1	1
SJM83	1	1	hsp Amerind	Amerind	Western	6	Out	1	EPIYA A, B, C	1	1	1	1	1	1	1	1
SJM92	1	1	hsp Amerind	Amerind	Eastern	3	In	1	EPIYA A, B, C	1	1	1	1	1	1	1	1
SJM93	1	1	hp Europe	Modern	Western	6	In	1	EPIYA A, B, C	1	1	0	1	1	1	1	0
SJM10	1	1	hsp Amerind	Amerind	Western	6	In	1	EPIYA A, B, C	1	1	0	1	1	1	1	1
SJM12	1	1	hsp Amerind	Amerind	Eastern	4	In	1	EPIYA A, B, C	1	1	0	1	1	1	1	1
SJM148	1	1	hp Europe	Modern	Western	6	In	1	EPIYA A, B, C	1	1	1	1	1	1	1	1
SJM14	1	1	hp Europe	Modern	Western	7	Out	1	EPIYA A, B, C	1	1	0	1	1	1	1	1
SJM179	1	1	hp Europe	Modern	Western	6	In	1	EPIYA A, B, C	1	1	1	1	1	1	1	1
SJM180	1	1	hp Europe	Modern	Western	6	In	1	EPIYA A, B, C	1	1	0	1	1	1	1	1
SJM189	1	1	hp Europe	Modern	Western	6	In	1	EPIYA A, B, C	1	0	1	1	1	1	1	1
SJM18	1	1	hp Europe	Modern	Eastern	4	In	1	EPIYA A, B, C	1	1	0	1	1	1	1	1
SJM19	1	1	hp Europe	Modern	Eastern	4	Out	1	EPIYA A, B, C, C	1	0	1	1	1	1	1	1
SJM1	0	1	hp Europe	Modern	Western	6	In	1	EPIYA A, B, C	0	0	0	1	1	1	1	1
SJM20	1	1	hp Europe	Modern	Western	6	In	1	EPIYA A, B, C	1	1	1	1	1	1	1	1
SJM21	1	1	hp Europe	Modern	Western	6	In	1	EPIYA A, B, C	1	1	0	1	1	1	1	1
SJM23	1	1	hsp Amerind	Amerind	Western	6	In	1	EPIYA A, B, C	1	1	0	1	1	1	1	1
SJM37	1	1	hp Europe	Modern	Western	6	In	1	EPIYA A, B, C	1	1	1	1	1	0	1	1
SJM38	1	1	hp Europe	Modern	Western	6	In	1	EPIYA A, B, C	1	0	1	1	1	0	1	1
SJM41	1	1	hp Europe/WAfrica	Modern	Eastern	2	Out	1	EPIYA A, B, C	1	1	1	1	1	1	1	1
SJM45	1	1	hp Europe	Modern	Western	8	In	1	EPIYA A, B, C, C	1	1	1	1	1	1	1	1
SJM50	1	1	hp Europe	Modern	Eastern	5	Out	1	EPIYA A, B, C, C	1	1	1	1	0	1	1	1
SJM53	0	1	hp Europe	Modern	Western	6	In	1	EPIYA A, B, C	0	0	1	1	1	1	1	1
SJM63	1	1	hp Europe	Modern	Western	6	Out	1	EPIYA A, B, C	1	1	0	1	1	1	1	1
SJM69	1	1	hp Europe	Modern	Western	6	In	1	EPIYA A, B, C	1	1	0	1	1	1	1	1

***B) Other Strains***																	

Spain (HUPB76)	1	1	hp Europe	Modern	Western	9	In	1	EPIYA A, B, C, C	1	0	1	1	1	0	1	1
Japan (HU178)	1	1	hp East Asia	East Asia	Eastern	4	In	1	EPIYA A, B, D	1	1	1	1	1	1	1	1
Africa (10)	1	1	hp S Africa	Modern	Western	6	In	1	EPIYA A, B, C	0	1	1	1	1	1	1	1
India (MS8)	1	1	hp Europe	Modern	Western	6	In	1	EPIYA A, B, C	1	1	1	1	1	1	0	0
